# Effect of using biodegradable film constituting red grape anthocyanins as a novel packaging on the qualitative attributes of emergency food bars during storage

**DOI:** 10.1002/fsn3.3951

**Published:** 2024-01-25

**Authors:** Reza Yekta, Arasb Dabbagh Moghaddam, Hedayat Hosseini, Anousheh Sharifan, Saeed Hadi, Seyyed‐Javad Hosseini‐Shokouh

**Affiliations:** ^1^ Infectious Diseases Research Center Aja University of Medical Sciences Tehran Iran; ^2^ Department of Public Health and Nutrition, Faculty of Medicine Aja University of Medical Sciences Tehran Iran; ^3^ Department of Food Science and Technology, National Nutrition and Food Technology Research Institute, Faculty of Nutrition Science and Food Technology Shahid Beheshti University of Medical Sciences Tehran Iran; ^4^ Department of Food Science and Technology, Science and Research Branch Islamic Azad University Tehran Iran

**Keywords:** anthocyanin, biodegradable film, emergency food bar, fat rancidity, hardness, red grape pomace

## Abstract

This study presents a novel packaging film based on whey protein isolate/κ‐carrageenan (WC) with red grape pomace anthocyanins (RGA) to investigate its impact on some qualitative attributes of emergency food bars (EFBs) for 6 months at 38°C. Increasing the RGA dose in WC films from 5% (WCA5) to 10% (WCA10) reduced hydrogen bonding between polymers and polymer homogeneity in the matrix according to FTIR and SEM. Tensile strength slightly declined in WCA5 from 7.47 ± 0.26 to 6.97 ± 0.12, while elongation increased from 27.74 ± 1.36 to 32.36 ± 1.25% compared to WC film. The maximum weight loss temperature (T_M_) increased by incorporating 5 wt% RGA from 182.95°C to 244.36°C, whereas T_M_ declined to 187.19°C in WCA10 film. WVP and OTR slightly changed in WCA5 (from 7.83 ± 0.07 and 2.57 ± 0.18 to 8.41 ± 0.03 g H_2_O.m/m^2^.Pa.s × 10^−9^ and 1.79 ± 0.32 cm^3^ O_2_/m^2^.d.bar, respectively), but significantly impaired in WCA10 compared to WC film. WCA5 and WCA10 films had high AA%, 68.77%, and 79.21%, respectively. WCA10 film presented great antimetrical properties against *Staphylococcus aureus* with an inhibition zone of 6.00 mm. The light transmission of RGA‐contained films in the UV spectrum was below 10%. The WCA5 film effectively restrained moisture loss and hardness increment until the end of the storage period, which were 14.33% and 28.76%, respectively, compared to day 0. Antioxidant films provided acceptable resistance against oxidation to EBF treatment. Sensory panels scored WCA5 and WCA10 higher in overall acceptance with 5.64 and 5.40 values, respectively, while complaining about the hardness of OPP treatment. The results of this investigation demonstrated that incorporating RGA, preferably 5 wt%, into WC‐based film effectively improved the qualitative properties of EFB during the 6‐month shelf life. This film might be a promising alternative for packaging light and oxygen‐sensitive food products.

## Graphical abstract

In the current investigation, a potent antioxidant film was prepared by incorporating red grape anthocyanin (RGA) into a film made of whey protein isolate and k‐carrageenan. Excessive concentrations of RGA, such as 10%, might lead to the deterioration of the mechanical and barrier properties of the film confirmed by SEM. During a 6‐month shelf life period, the film containing 5% RGA was found to effectively reduce fat rancidity in emergency food bars.

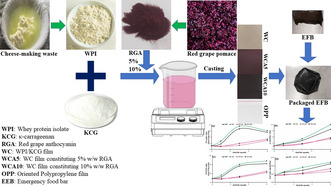

## 1 INTRODUCTION

Food packaging has evolved beyond being a mere container for food. It now plays an active role in improving the quality and longevity of the food it contains. This is achieved by integrating bioactive compounds in the packaging material, which offer a range of beneficial effects (Singh et al., 2022). Active food packaging can be classified into several categories based on the function of the additive. Antioxidant additives prevent the formation of free radicals and delay oxidation reactions, while antimicrobial additives inhibit the growth of microorganisms responsible for food spoilage and contamination (Baghi et al., 2022; Tkaczewska et al., 2023). Using bioactive compounds with both antioxidant and antimicrobial properties in edible films is becoming increasingly popular. These compounds can be sourced from plants, by‐products, and agri‐food waste, thus reducing food waste and adding value to the food industry (Baghi et al., 2022; Monção et al., 2022; Singh et al., 2022).

Emergency food bars (EFBs) are a highly nutritious food source that can be consumed in various situations, including by athletes or individuals affected by natural disasters or disease outbreaks, e.g., COVID‐19 (Dabbagh Moghaddam et al., 2019; Norouzbeigi, Vahid‐Dastjerdi, Yekta, Farhoodi, & Mortazavian, 2021; Norouzbeigi, Yekta, Vahid‐Dastjerdi, Keyvani, Ranjbar, Shadnoush, Khorshidian, et al., 2021; Norouzbeigi, Yekta, Vahid‐Dastjerdi, Keyvani, Ranjbar, Shadnoush, Yousefi, et al., 2021; Yekta et al., 2021). The ideal EFB should be healthy, tasty, easily distributed, and have a shelf life of 6 months at 38°C (Brisske et al., 2004). EFBs are designed with a low moisture content and a_w_ nature, which protects them against microbial spoilage (Dabbagh Moghaddam et al., 2019; Ghorbani et al., 2021; Mohammadian, Moghaddam, Almasi, et al., 2021; Sheibani et al., 2018). Packaging materials must safeguard the bars from oxygen and water vapor to ensure freshness. Common materials like oriented polypropylene (OPP) and metalized polymers are used for packaging EFBs (Galić et al., 2009). Innovations in sustainable packaging, such as edible films and coatings, are also being developed to increase shelf‐life. Palatability and safety are the primary concerns regarding the quality of EFBs (Monção et al., 2022). Textural deterioration and chemical spoilage, like fat rancidity, can negatively impact the product's taste and safety.

Antioxidants are employed to prevent the oxidation of fats and oils. While synthetic antioxidants such as tert‐butyl hydroquinone (TBHQ), butylated hydroxyanisole (BHA), and butylated hydroxytoluene (BHT) are prevalent, they may have undesirable side effects (Felter et al., 2021; Husøy et al., 2019; Khezerlou et al., 2022; Lourenço et al., 2019). As a result, natural antioxidants sourced from herbs, vegetables, fruits, and spices are currently being examined as a viable alternative (Lourenço et al., 2019). Red grapes, in particular, boast high levels of phenolic compounds, making them an excellent source of antioxidants (Magrone et al., 2020). Not only have they been utilized in intelligent packaging and biosensors to indicate freshness, but they also hold great promise as a natural antioxidant (Chi et al., 2020; Golasz et al., 2013; Ma & Wang, 2016; Yekta, Abedi‐Firoozjah, et al., 2023; Yoshida et al., 2014).

To the best of our knowledge, Iran is a prominent global producer of grapes, contributing around 2 out of 77 million tons in 2020 (Roshani Bakhsh et al., 2019). Interestingly, the waste produced during grape juice and concentrate production, known as red grape pomace (RGP), is abundant in polyphenols (Makris et al., 2016). While fruits, vegetables, and herbs are renowned for their natural antioxidant properties, these properties can be lost during cooking and pasteurization (Khanal et al., 2010). This is where edible films come into play, serving as a carrier for these antioxidants. Innovative packaging films have been created from natural materials, including polysaccharides (Mohammadi et al., 2023; Yekta et al., 2020), lipids (Yousuf et al., 2022), and proteins (Hadidi et al., 2022). These films have been made from a variety of sources such as zein (Rodríguez‐Félix et al., 2022), sweet potato starch (Choi et al., 2022), starch‐gelatin (Cheng et al., 2023; Dakhili et al., 2024), cellulose (Zhang, Li, et al., 2023), chitosan (Dordevic et al., 2021), basil seed gum/chitosan (Nadi et al., 2023), levan/pullulan/chitosan (Gan et al., 2022), and pullulan polysaccharide/xanthan gum (Zheng et al., 2023). Additionally, they have been enhanced with grape extracts (Dordevic et al., 2021; Zhang, Li, et al., 2023; Zheng et al., 2023), red cabbage anthocyanin (Nadi et al., 2023), betalains (Rodríguez‐Félix et al., 2022), ε‐polylysine (Gan et al., 2022), parsley and blueberry extracts (Dordevic et al., 2021), to make them customized for specific food applications, such as packaging strawberries, apples, and meat products. These bio‐based films help preserve the natural flavors and freshness of food products, while extending their shelf life.

There has been growing interest in developing active and intelligent packaging films using biopolymers containing anthocyanins in recent years (Cheng et al., 2023; Choi et al., 2022). These films can help preserve food quality and prolong shelf life. Anthocyanins have potential antioxidant and antimicrobial properties, making them ideal for use in food packaging (Mushtaq et al., 2018; Wang et al., 2019). These films can be produced through casting or extrusion methods. Previous research has shown that phenolic compounds have a significant binding affinity to proteins, particularly the proline amino acid group of proteins (Qin et al., 2019). These interactions can result in different structural modifications. Whey protein, milk casein, and wheat gluten have been used to make food packaging films (Kandasamy et al., 2021). This protein is abundant and important in food production, making it a popular choice for food formulations (Meganaharshini et al., 2023), films (Al‐Hilifi et al., 2023; Rossi‐Márquez et al., 2023), nanofibrils (Hasan et al., 2023), and micro/nano‐carriers for bioactive ingredients (Esmaeili et al., 2022; Hosseiniyeh et al., 2023). Mixing various ingredients with distinct characteristics to create synergistic effects is a viable method of producing new blend films. For example, Cheng et al. (2023) developed a highly hydrophobic edible packaging film by combining starch and gelatin and incorporating three natural waxes using the extrusion‐blowing procedure. Containing natural waxes such as carnauba wax, candelilla wax, and beeswax can improve the water vapor permeation rate, water contact angle, and thermal resistance of the film. Incorporating levan, pullulan, and grape seed extract into films has also been shown to improve their properties (Gan et al., 2022). For instance, a chitosan‐based film was reinforced with levan, pullulan, and an antimicrobial agent, ε‐polylysine, to be used as strawberry coatings. The resulting film demonstrated a lower oxygen vapor permeation rate and water solubility while increasing the film's elongation. The developed film also improved the qualitative properties of strawberries during storage (Gan et al., 2022). Zheng et al. (2023) incorporated grape seed extract (GSE) into a pullulan/xanthan gum (PXG)‐based film and investigated the impact of the active film on the physicochemical properties of fresh‐cut apples. The resulting film extended the shelf life of fresh‐cut apples by improving their antibacterial, antioxidant, mechanical, UV barrier, and water resistance properties. Furthermore, edible films can maintain bioactive components such as natural antioxidants and antimicrobials, making them an excellent vessel for transporting bioactive ingredients into food products. Previous studies have also suggested the potential of incorporating κ‐carrageenan into whey protein to reinforce the film properties (Sogut et al., 2019, 2022). In conclusion, developing active and intelligent packaging films using biopolymers containing anthocyanins is a promising field with potential benefits for the food industry.

Therefore, the objectives of the current study were to:

1. Investigate the biocompatibility of RGA to the WPI/κ‐carrageenan (WC) matrix.
2. Analyze the effect of incorporating red grape anthocyanin (RGA) extracted from grape pomace at different doses on the physicochemical and structural properties of the composite film.
3. Fabricate an edible packaging film with enhanced antioxidant, UV‐blocking, and oxygen permeation barrier properties to avoid EFB oxidation.
4. Monitor the effect of antioxidant film on the qualitative parameters of EFB‐coated treatments during the shelf life compared to OPP‐coated ones.

To the best of our knowledge, no study has been conducted to investigate the impact of an antioxidant packaging film on the shelf life‐dependent parameters of EFB.

## 2 MATERIALS AND METHODS

### 2.1 Materials

Red grape pomace was from the *Shiraz* cultivar (*Vitis vinifera*), obtained from a local semi‐industrial grape juice producer (Arak, Iran). Ethanol was supplied by Bidestan Co (Qazvin, Iran). Food‐grade whey protein isolate (protein content ~ 94.3%) was purchased from Arla® Foods Co (Viby, Denmark). 2,2‐diphenyl‐1‐picrylhydrazyl (DPPH) and κ‐carrageenan (reagent grade) were provided by Sigma‐Aldrich® Co (St. Louis, USA). Glycerol, calcium chloride (CaCl_2_), sodium chloride (NaCl), magnesium nitrate [Mg(NO_3_)_2_], and all other chemicals were of analytical grade. Mueller–Hinton Broth (MHB), Mueller–Hinton agar (MHA), and Sabouraud dextrose agar (SDA) were purchased from Merck Co (Darmstadt, Germany). *Escherichia coli* ATCC 25922, *Staphylococcus aureus* ATCC 25923, and *Candida albicans* ATCC 10231 were provided by the Iranian Research Organization for Science and Technology (Tehran, Iran).

### 2.2 Methods

#### 2.2.1 Red grape anthocyanin isolation

Red grape pomace was dried at 25°C for 48 h immediately after receiving it at the laboratory, then milled using a miller blender (Hamilton, USA) and stored in a refrigerator at 4°C. According to Makris et al. (2008), the anthocyanin extraction was carried out with minor modifications (Makris et al., 2008). Briefly, 450 g of the dried was incorporated into a 3 L beaker containing 1000 mL ethanol solution (85.5%, v/v) and placed in a dark place to macerate for 30 min. Then, the beaker was placed on a heater stirrer, and 1500 mL of the ethanol solution was added and mixed for 15 min. Afterward, the mixture was centrifugated at 4500 *g* for 10 min, and the supernatant was concentrated and then lyophilized (Crist Alpha 2‐4 LD plus). The dried extract was tested for the measurement of total phenolic compounds (according to Folin–Ciocalteu assay and gallic acid was used as standard), anthocyanins (malvidin‐3‐glucoside was used as standard), and DPPH radical scavenging activity (Ju & Howard, 2003), which were 5361.6 ± 18.3 mg/100 g powder, 282.2 ± 7.8 mg/100 g powder, and 87.19 ± 0.76%. Table 1 demonstrates the physicochemical analysis of WPI, KCG, and RGA.

**TABLE 1:** Physicochemical properties of WPI, KCG, and RGA.

**WPI**	
Protein (%)	94.3
Lactose (%)	≤0.09
Fat (%)	1.5
Moisture content (%)	5.1
Ash (%)	1.2
pH (5% solution W/V)	6.35
Bulk density (g/cm^3^)	0.45
Solubility index (mL)	0.1
Wettability (%/10 s)	80
**KCG**	
Viscosity (0.3% in H_2_O at 25°C) (mPa.s)	5–25
Solubility (g/L in hot H_2_O)	5
Gel strength (1.5% + 0.2% KCl 20°C) (g/cm^2^)	≥1300
Moisture (%)	8
Total Ash (%)	15–40
pH (1% solution W/V)	8–11
Sulfates (%)	15–40
**RGA (All values are mean ± SD)**	
Total phenolic compounds (mg/100 g powder)	5361.6 ± 18.3
Anthocyanins (mg/100 g powder)	282.2 ± 7.8
DPPH radical scavenging activity (%)	87.19 ± 0.76
Moisture content (%)	4.71 ± 0.55
Ash (%)	0.58 ± 0.07
pH (2%)	2.85 ± 0.01

#### 2.2.2 Film preparation

WPI/κ‐carrageenan film‐forming solution was prepared according to the Sogut et al. (2022) method with a few modifications. First, the solutions containing either whey protein (5 wt%) or κ‐carrageenan (1 wt%) were prepared by separately dissolving the specified concentration of the polymers in deionized water. The pH of the whey protein solution was adjusted to 8 via 2 N NaOH, then heated at 90°C for 20 min. Meanwhile, the κ‐carrageenan solution was prepared by heating at 80°C for 15 min. Afterward, 50% and 35 wt% (based on the dry weight of the polymers) glycerol were incorporated into the protein and polysaccharide‐containing solutions, and the temperature of the solutions cooled down to 40°C. Finally, the solutions were mixed at a ratio of 1:1 (v/v) and constantly mixed at 40°C for 15 min at 800 RPM, then poured onto a Teflon plate with a diameter of 14.5 cm, and surface area of 45.55 m^2^. For treatments containing RGA, 5% and 10 wt% of RGA were added prior to final mixing. The treatments were WC (a film without RGA), WCA5 (WC film containing 5% RGA), and WCA10 (WC film containing 10% RGA). The films were stored in a desiccator containing Mg (NO_3_)_2_ solution (RH: 54%) at 25°C.

#### 2.2.3 Film characterization

##### Antioxidant properties

The radical scavenging activity of the films was determined using the Brand‐Williams et al. (1995) procedure, which is based on the decolorizing of DPPH methanol solution (purple‐colored or bluish‐red) by an antioxidant agent (Shojaee‐Aliabadi et al., 2014). In summary, 25 mg of each treatment was constantly stirred to dissolve it in 5 mL of distilled water. 0.1 mL of this solution was mixed with 3.9 mL of DPPH methanol solution (0.1 mM). The obtained blend was placed in a dark place for 1 h at ambient temperature, and then the absorbance (A) was determined at 517 nm against the blank sample (methanol solution of DPPH). DPPH radical scavenging activity (%) was determined according to Equation 1:

(1) $$\text{DPPH scavenging activity}\;\left(\%\right)=\frac{\left(A_{\text{blank}}\right)-\left(A_{\text{treatment}}\right)}{A_{\text{blank}}}\times 100$$

##### Antimicrobial properties

The disc diffusion procedure was applied to evaluate the antimicrobial activities of WC, WCA5, and WCA10 against pathogenic microorganisms, including *S. aureus*, *E. coli*, and *C. albicans*. Stock cultures of the studied bacteria and fungi were grown in MHB and SDA at 35°C for 24 h and 23 ± 2°C for 48 h before the tests, respectively. Film samples were aseptically cut into 6 mm diameter discs and placed on plates containing MHA and SDA, previously seeded with 100 μL of an overnight broth culture containing approximately 10^8^ and 10^6^ CFU/mL of the test bacteria and fungi. The diameter of inhibition zones was measured in triplicate for each treatment after incubation at 37°C for 24 h (Dordevic et al., 2021).

##### Structural properties

ATR‐FTIR spectra of control and active films were analyzed by a Perkin‐Elmer FTIR spectrophotometer (Spectrum 1, USA) between 600 and 4000/cm range using a resolution of 4/cm. An average of 16 scans has been mentioned for each sample. The analysis was performed at ambient temperature.

##### Thermal properties

Thermogravimetry analysis (TGA) of the treatments was performed by a Perkin‐Elmer thermal analyzer (Pyris diamond, USA) in a range of 40 to 550°C at a heating rate of 10°C/min under 50 cm^3^/min constant nitrogen flow. Around 1 mg of each treatment was moved into an aluminum pan, and the empty aluminum pan was considered the reference. The derivative of TGA (DTG) Equation 2 was obtained by differentials of TGA values according to the following formula:

(2) $$\mathrm{DTG}=\frac{\text{the residual weight of sample}\;\mathrm{at}\;\text{time}\;\left(\left(t+∆t\right)-\left(t-∆t\right)\right)}{\text{the time interval for reading residual sample weight}\;\left(∆t\right)}\times 100$$

##### Physical properties

The thickness of each treatment was determined by taking the average of measurements obtained from 10 different points using a micrometer (Mitutoyo, Japan) with a precision of 0.001 mm.

The treatments' moisture content (MC) was determined using an oven‐drying method that involved drying films in an oven at 110°C until a constant weight (dry weight) was achieved. The MC was calculated for each treatment by measuring three replications and applying the equation below:

(3) $$\mathrm{MC}\;\left(\%\right)=\frac{\left(\text{Initial weight}\right)-\left(\mathrm{Dry}\;\text{weight}\right)}{\text{Initial weight}}\times 100$$

In order to calculate the water solubility (WS) of films, three samples of each treatment were cut into rectangle shapes (1 × 3 cm), and each was moved into a beaker containing 50 mL distilled water after weighing (A&D, Japan). The samples were submerged under continuous and gentle agitation at ambient temperature for 24 h and then filtered. Undissolved film pieces were put in an aluminum pan and moved into an oven at 110°C to dry to a constant weight. The initial dry weight of each sample was determined as well. WS was calculated according to the following equation:

(4) $$\mathrm{WS}\;\left(\%\right)=\frac{\left(\text{Initial}\;\mathrm{dry}\;\text{weight}\right)-\left(\mathrm{Dry}\;\text{weight of unsoluble material}\right)}{\text{Initial}\;\mathrm{dry}\;\text{weight}}\times 100$$

##### Barrier properties to gases

This study conducted a series of experiments to determine the water vapor permeability of film samples (Shojaee‐Aliabadi et al., 2014). Each cup, which had a surface area of 0.00287 m^2^ and contained approximately 50 g of anhydrous calcium chloride (0% RH, assay cup), was sealed with film samples in triplicate. The sealed cups were then placed in desiccators that contained a saturated sodium chloride solution at 75% RH. The water vapor partial pressure was determined to be 1753.55 Pa, indicating the RH differences between the inner and outer atmosphere of cups. The weight of cups was consecutively recorded every hour for a 12 h period with a precision of 0.001 mm. WVP and water vapor transmission rate (WVTR) were measured according to these formulas:

(5) $$\text{WVTR}=\frac{\text{The weight gain versus time slope}\;\left(g/s\right)\;}{\text{Exposed surface area of film}\;\left(m^{2}\right)}$$

(6) $$\mathrm{WVP}=\text{WVTR}\times \frac{\text{Film thickness}\;\left(m\right)}{\text{Difference of pressure}\;\text{between the inner and outer sides of}\;\mathrm{cup}\;\left(\mathrm{Pa}\right)}$$

In order to measure the transmission rate of O_2_ (OTR) through film treatments, a gas permeability tester (Coesfeld model GDP‐C, Germany) was used. The samples were placed in a chamber and sealed as well at ambient temperature. The vacuum was applied in the whole system for 5 min before pure O_2_ (99.9%) was administered on one side. The gradient of O_2_ in the testing machine cell was 0.1 Pa, and the OTR values (carried out in triplicate) in cm^3^/m^2^.d.bar were calculated according to this formula:

(7) $$\mathrm{OTR}=\frac{\left(\text{Mean}\;\mathrm{OTR}\;\right)\times \left(\text{Film thickness}\right)}{\text{The oxygen gradient in testing machine cell}}$$

##### Mechanical properties

Tensile strength; TS, elongation at break; EAB, and Yong's modulus (YM) of the film treatments were computed by a Testometric Machine M350‐10CT (Testometric Co., Ltd., Rochdale, Lancs., England) at 25°C according to ASTM standard method D882 (ASTM D 882–02, 2002). Before testing, five rectangular strips were cut from each film sample with a dimension of 10 × 1.5 cm, and the initial cross‐head speed and grip spacing were set at 50 mm/min and 50 mm, respectively. TS, EAB, and YM values were computed as follows:

(8) $$\mathrm{TS}=\frac{\text{Maximum force}}{\left(\text{Film width}\right)\times \left(\text{Film thickness}\right)}$$

(9) $$\mathrm{EB}\;\left(\%\right)=\frac{\left(\text{maximum length before breaking}\right)-\left(\text{Initial length}\right)}{\text{Initial length}}\times 100$$

(10) $$\mathrm{YM}=\frac{\mathrm{TS}}{\mathrm{EB}\%}\times 100$$

##### UV Barrier properties and transparency

Strips of film measuring 0.5 × 3 cm were placed into quartz cuvette cells. The UV–vis spectrophotometer (Perkin Elmer model Lambda 2, Germany) was used to read the light transmission in wavelengths ranging from 200 to 800 nm. For the RGA, a 0.15% solution was prepared with deionized water. The measurements were performed five times, and the results were presented as the mean. In addition, the transparency at 600 nm (T600) was calculated using the following formula (Monjazeb Marvdashti et al., 2017):

(11) $$\text{Transparency}=\frac{{\text{logT}}_{600}}{\text{Thickness}}$$

##### Microstructure

Scanning electron microscope (VEGA 2, Tescan, Czech Republic), SEM, was used to analyze the surface and cross‐sectional microstructures of the film treatments. Each formulation was placed onto a copper stub, stabilized, and coated with platinum (SC 7620, England). All film samples were scanned under vacuum conditions (10^−5^ Pa) with a 30 kV accelerating voltage. The films were frozen using liquid nitrogen to assess the cross‐section area and then fractured. The films were examined at a magnification of ×2500 for surface microstructure and ×500 for cross‐sectional microstructure.

#### 2.2.4 Emergency bar preparation

Emergency food bars were prepared according to Ghorbani et al. (2021) method with a few modifications in the form of 50 g solid bars: 11 g shortening (fat content: 70%, and constituted 0.01% ascorbyl palmitate) was placed in an oven at 110°C to melt then 0.5 lecithins was incorporated, and other ingredients including 25 g wheat flour (protein content: 9.5%), 5 g skim milk powder (protein content: 35.5%), 7 g sugar, vanilla (0.5 g), cocoa powder (0.5 g), coconut powder (0.75 g), DSM vitamins/minerals premix (3.5 g), and salt (0.25 g) were added to melted shortening and mixed thoroughly, and 5 mL of water was added to the mixture. Using aluminum foil, a 54 g semisolid mixture was molded in 4.4 × 7.6 cm dimensions. Finally, the baking process was done in the oven at 150°C for 20 min. The trays containing EFBs were left in a clean place at ambient temperature for 15 min to cool down. Afterward, EFBs coated by WC, WPA5, WCA10 films, and an OPP film which was also used as reference (thickness: 12.2 ± 0.3 μm, WVP: 8 g.m/m^2^.day.Pa, and OTR: 15.1 cm^3^/m^2^.day.bar).

#### 2.2.5 Characterization of the product during shelf life period

##### Water activity (a_w_)

Water activity of EFB treatments was determined by a Ro‐tronic A2 hygrometer (Ro‐tronic AG, Bassersdorf, Switzerland) immediately after production and the months 1, 3, and 6 of storage. Before loading the hygrometer cup, EFBs were crushed into small pieces.

##### Moisture content

Moisture content of EFB treatments was determined by a thermogravimetric method immediately after EFB preparation and in months 1, 3, and 6 after production using digital moisture analyzer equipment (RADWAG® Co, Poland). Samples were milled in a miller blender, and 5 g of each treatment was dried until a constant weight was reached at 130°C.

##### Hardness

The hardness of EFBs was computed on day 0 and months 1, 3, and 6 using a texture analyzer (Testometric M350‐10CT, United Kingdom), and the records were collected via computer software. A 50 kg load cell was set up, and treatments were placed on the platform. Samples were tested by a needle probe with a penetration depth of 1 mm operating at a pre‐test speed of 1 mm/s, a test speed of 0.5 mm/s, a post‐speed of 3 mm/s, and a distance of 5 mm. Five EFBs were tested per treatment to ensure accurate results.

##### Fat oxidation

The oxidative condition of fats in EFB treatments was measured according to AOCS Cd 8b‐90 and Cd 18‐90 methods (AOCS, 1993) and AACC 02–01 method (AACC, 2000). These qualitative tests included peroxide value (PV), p‐anisidine value (p‐AV), TOTOX value (=2 × PV + p‐AV), and acid value. The analysis was performed on the oil extracted from the EFB samples immediately after EFB production and during the shelf life period at months 1, 3, and 6. The fat extraction method was based on cold extraction with n‐hexane, according to Hallabo's (1977) description. It is worth mentioning that PV and p‐AV for shortening were 0.46 meq O/kg and 0.11, respectively.

##### Sensory evaluation

Emergency food bars treatments were assessed for their sensory characteristics, including color, texture, taste, and overall acceptance, on a nine‐point hedonic scale (1, dislike extremely to 9, like extremely). Twenty‐five semi‐trained sensory panels (64% male and 36% female, aged 19 to 56) volunteered to evaluate the treatments. The sensory panels were invited to consider the sensorial characteristics of EFBs immediately after production and the first and last months of storage.

#### 2.2.6 Statistical analysis

All experiments were conducted in triplicate. To evaluate the impact of RGA incorporation in different doses on the characteristics of the WC film, a one‐way analysis of variance (ANOVA) and Duncan's multiple range test were conducted using IBM SPSS software V.26 (IBM SPSS Inc., Chicago, IL, USA). A two‐way ANOVA was employed to investigate the influence of storage period, treatments, and interactions on the physicochemical properties of EFBs. The General Linear Model (GLM) was used to compare the scores of different sensory attributes among the treatments and storage periods. The data was presented as mean ± standard error, and statistical significance was accepted at a probability of *p* < .05 for all experimental data.

## 3 RESULTS AND DISCUSSION

### 3.1 Fourier transform infrared analysis

**FIGURE 1:**
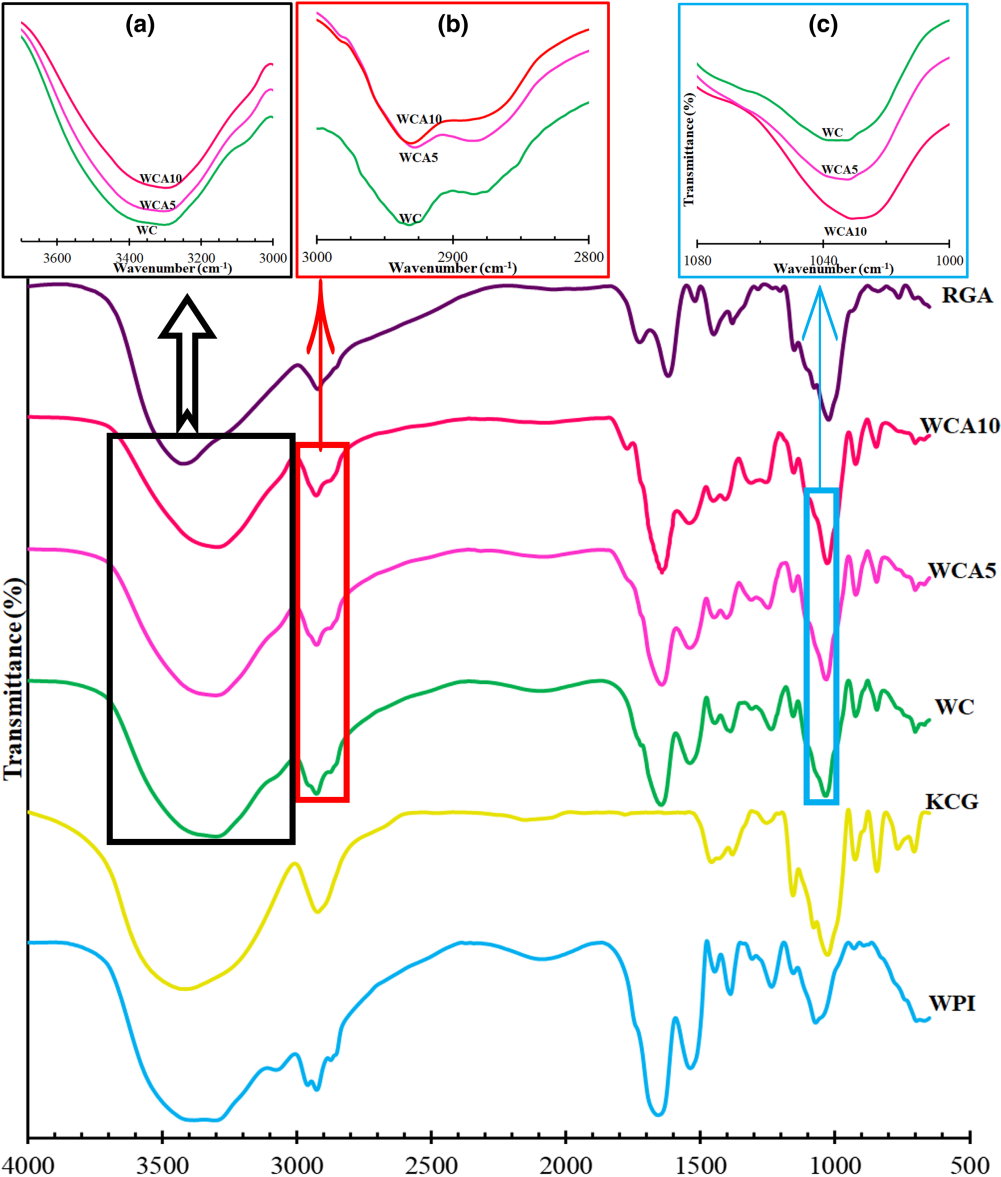
ATR‐FTIR spectra of WC and RGA‐contained films. Changes in the band's intensity at the region of 3700 to 3000/cm (a), 2925/cm (b), and at the region of 1060 to 1020/cm (c) by increasing RGA concentration from 0 to 10% in WCA film. Abbreviations: RGA, anthocyanins extracted from red grape pomace; WC, Whey protein isolate (WPI)/κ‐carrageenan film (KCG); WCA5, WC film constituting 5 wt% RGA; WCA10, WC film constituting 10 wt% RGA.

Fourier transform infrared analysis is an analytical approach to closely examine the film's structure changes. Figure 1 demonstrates FTIR curves corresponding to WPI, KCG, WC, WCA5, WCA10 films, and RGA. Absorption peaks at 3700 to 3000/cm, 3000 to 2800/cm, and 1200 to 750/cm corresponded to N—H and free O—H groups, C—H stretching vibrations, and the vibration bonds of C—O and C—C, respectively (Chi et al., 2020; Gilbert et al., 2005; Ili Balqis et al., 2017; Xu et al., 2021). Signals related to Amid I (C=O and C—N stretching vibrations), Amid II (N—H groups), and Amid III (N—H and C—N stretching vibrations) appeared at wavenumber ranges of 1700–1600/cm, 1550–1400/cm, and 1350–1200/cm, respectively (Agudelo‐Cuartas et al., 2021; Dong et al., 2018; Yekta, Assadpour, et al., 2023). The spectrum range between 1480 and 1200/cm was also believed to be the fingerprint region for protein‐based films (Dong et al., 2018). Moreover, the so‐called fingerprint region for this polysaccharide is known to be in the range of 1500–800/cm (Ili Balqis et al., 2017). The signals that appeared at 844, 926, and 1156/cm were possibly aroused from the vibrations of galactose‐4‐sulfate (C—O—S), 3,6‐anhydrogalactose (C—O—C), and sulfate ester (O=S=O), respectively (Ili Balqis et al., 2017). Additionally, the signal at 1028/cm might have originated from the stretching vibrations of the C—O—C glycosidic bond (Liu et al., 2021). RGA demonstrated the characteristic signals of anthocyanins at 3404, 1617, 1451, and 1023/cm, arousing from stretching vibrations of hydroxyl groups, stretching vibrations of CC = aromatic rings, angular deformation of C—O groups in phenols, and stretching vibrations of C—O—C band within anhydroglucose ring in flavonoids' structure (Liu et al., 2021).

In the blend film (WC), the signal intensity related to free hydroxyl groups remarkably decreased, possibly due to the formation of hydrogen bonds between —OH groups that existed in the structure of both polymers. Moreover, the latest interaction between —OH groups in WPI and sulfur ester groups of carrageenan might also occur. This observation was in line with that of Sogut et al. (2019), who claimed that the main functional groups of KCG assisted in the interaction with WPI were sulfate and hydroxyl groups. Furthermore, the intensity of the signal at 1648/cm decreased in the WC treatment compared to WPI film, which could be ascribed to hydrogen binding (Gilbert et al., 2005). The latest signal is characteristic of amide groups that participated in the extended β‐sheet structure, which possibly enhanced intermolecular interactions among whey proteins and other compounds via the exposed sulfhydryl functional group after heat denaturation of the protein (Nicolai et al., 2011). Additionally, hydrophobic interactions could contribute to maintaining the WC film's structure (Sogut et al., 2019). The incorporation of RGA at 5% and 10 wt% concentration did not considerably change the peak position in comparison with the treatment without RGA. Ma and Wang (2016) also reported no significant alteration in the band position of Tara gum/nanocrystalline cellulose film after the incorporation of red grape extract, suggesting the entrapment of the extract in the film matrix. However, the band's intensity at the region of 3700 to 3000/cm gradually decreased by increasing the incorporation dose of RGA from 0 to 10% (Figure 1a), possibly due to the higher moisture content of RGA‐containing films (Table 2). The strength of the signal associated with hydroxyl groups may reduce as the moisture content rises in films because of the development of hydrogen bonds between water molecules and the free hydroxyl groups in the film (Calixto et al., 2019). Since anthocyanins are hydrophilic compounds, they enter more water molecules into the film matrix, and the water molecules connect to the molecular network of the film, resulting in the hydroxyl groups becoming less polar and the O‐H bond becoming stronger (Calixto et al., 2019). As a result, this can cause a decline in the signal intensity connected to hydroxyl groups in the IR spectrum (Calixto et al., 2019). Furthermore, hydrogen bonds might form due to the interaction between functional groups of anthocyanins and the polymer matrix of the film (Pourjavaher et al., 2017). The reduction of the intensity of the signal at 2925/cm by increasing RGA concentration from 0 to 10% (Figure 1b) could be considered another proof of the interaction between RGA and polymeric matrix, possibly due to the creation of new bonds between aromatic structures of anthocyanin and C—H containing groups in the film matrix (Figure 1c). Electrostatic interactions between cationic anthocyanin and anionic areas of both polymers, WPI and KCG, might also be formed in the film's matrix (Sogut et al., 2022). More to the point, the oxygen molecule in the central pyran ring is positively charged (Tsao & Mccallum, 2009). The signal intensity at 1032/cm sharply increased by incorporating 5% and 10% RGA into WC film, possibly due to abundant C—O—C stretching bonds in flavonoids (Liu et al., 2021).

**TABLE 2:** Mechanical properties of WC and RGA‐contained films^†^.

Film	Thickness (μm)	Tensile strength (MPa)	Elongation at break (%)	Yong's modulus (MPa)
WC	254 ± 9^b^	7.47 ± 0.26^a^	27.74 ± 1.36^b^	157.40 ± 23.02^a^
WCA5	282 ± 15^a^	6.97 ± 0.12^b^	32.36 ± 1.25^a^	137.33 ± 27.39^ab^
WCA10	303 ± 25^a^	6.61 ± 0.15^c^	32.26 ± 0.90^a^	128.63 ± 16.56^b^

*Note*: Different letters in the same column indicate significant differences between treatments (*p* < .05).

Abbreviations: RGA, anthocyanins extracted from red grape pomace; WC, Whey protein isolate/κ‐carrageenan film; WCA10, WC film constituting 10 wt% RGA; WCA5, WC film constituting 5 wt% RGA.

^†^ All values are mean ± SD.

### 3.2 Mechanical properties of films

Table 1 provides the data related to the mechanical properties and thickness of films. The thickness of active films containing RGA increased significantly from 254 to 282 μm compared to the control treatment (*p* < .05). Further increment in the RGA concentration (from 5% to 10%) did not significantly change the thickness. On the contrary, Ma and Wang (2016) did not observe any significant change in the film's thickness upon incorporating up to 15% grape skin extract (GSE).

Tensile strength of the film decreased by 6.69% and 11.51% with the incorporation of 5% and 10% RGA, respectively, and the higher the RGA concentration, the lower the TS. The latest outcome might be due to the negative impact of RGA molecules on the WPI‐WPI, KCG‐KCG, and WPI‐KCG interactions in a way that the mobility of the polymer chains in the film's matrix increased, leading to increasing EB (Ma & Wang, 2016). YM showed an alteration trend similar to TS.

### 3.3 Physical properties of films

The results of the MC and WS of the film treatments are demonstrated in Table 2. These parameters are indications for predicting the affinity of edible films toward water molecules (Monjazeb Marvdashti et al., 2017). More to the point, increasing these parameters might lead to increasing the water affinity of films (Monjazeb Marvdashti et al., 2017). Both properties significantly increased by adding RGA except for the MC of WCA5 film (*p* < .05). Furthermore, 36.77% and 43.01% increments in MC and WS of films were observed upon adding 10% RGA. This could be attributable to declining polymer‐polymer interactions in the film matrix after RGA incorporation (Ma & Wang, 2016). The latest phenomena increased by increasing RGA incorporation concentration. In other words, the amount of free volume in the film matrix increased by RGA incorporation. Consequently, there would be more room for water molecules to remain in the matrix of films.

### 3.4 Barrier properties of films against water vapor and oxygen molecules

The permeability of films to water vapor and oxygen molecules is shown in Table 3. The WVP of films significantly increased by 7.41% and 37.04%, adding 5% and 10% RGA, respectively. The WVP of the control treatment (=7.83 × 10^−9^ g.m/m^2^.Pa.s) was lower than that reported by Sogut et al. (2022), possibly due to the differences in the volume of the film‐forming solution that was poured in the Teflon plate. Moreover, the thickness of the control film (254 μm) in this study was higher than that of the latest study (92 μm). Generally, it is widely believed that the higher the thickness of a film, the lower the permeation rate (Gonz et al., 2019; Abdel Khafar et al., 2020; Ngo et al., 2020). However, the decrementation of intermolecular interactions in the film matrix caused by RGA incorporation might increase the permeability of films to water vapor molecules.

**TABLE 3:** Physical and barrier properties of WC and RGA‐contained films^†^.

Film	MC (%)	WS (%)	WVP (g.m/m^2^.Pa.s × 10^−9^)	OTR (cm^3^/m^2^.d.bar)
WC	22.51 ± 1.35^c^	30.11 ± 0.71^c^	7.83 ± 0.07^c^	2.57 ± 0.18^b^
WCA5	24.18 ± 0.59^bc^	32.65 ± 0.47^b^	8.41 ± 0.03^b^	1.79 ± 0.32^c^
WCA10	36.77 ± 1.04^a^	43.06 ± 1.53^a^	10.73 ± 0.11^a^	6.22 ± 0.47^a^

*Note*: Different letters in the same column indicate significant differences between treatments (*p* < .05).

Abbreviations: RGA, anthocyanins extracted from red grape pomace; WC, Whey protein isolate/κ‐carrageenan film; WCA10, WC film constituting 10 wt% RGA; WCA5, WC film constituting 5 wt% RGA.

^†^ All values are mean ± SD.

A different alteration trend was observed for OTR, in a way that the addition of 5% RGA into the WC film resulted in a significant decline (by 30.35%), possibly due to promoting the film's hydrophilicity as a result of incorporating hydrophilic anthocyanins (Khalil et al., 2018). However, an increment of 142% was observed for the film containing 10% RGA compared to the control treatment. The reduction of intermolecular interactions, particularly hydrogen bonding, creates a significant free volume, allowing oxygen molecules to permeate through the film more easily. Other studies also confirmed the detrimental effect of excessive incorporation doses of polyphenols extracted from grape and grapefruit seeds on the film's structure and barrier properties (Kanmani & Rhim, 2014; Rubilar et al., 2013).

### 3.5 Light barrier properties and transparency

**FIGURE 2:**
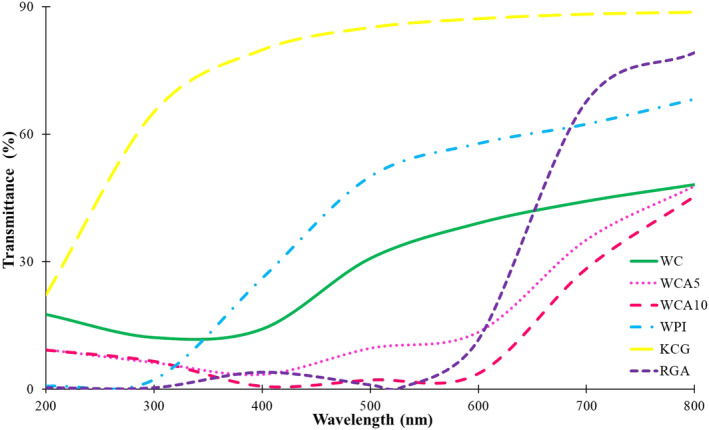
UV–Vis spectra of WC and RGA‐contained films. Abbreviations: RGA, anthocyanins extracted from red grape pomace; WC, Whey protein isolate (WPI)/κ‐carrageenan film (KCG); WCA5, WC film constituting 5 wt% RGA; WCA10, WC film constituting 10 wt% RGA.

UV‐light barrier properties of films are also a crucial factor that should be considered in designing packaging for food applications since it may devastate some light‐sensitive vitamins, initial or/and accelerate fat rancidity leading to the deterioration of the nutritional value and flavor of foods (Bekbölet, 1990). Figure 2 demonstrates the light transmission (LT) through films and RGA in wavelengths between 200 to 800 nm. WPI film and RGA demonstrated approximately zero transmission in the 200–300 nm range, UV‐B and UV‐C. LT gradually increased in the visible region (400–800) of WPI film. In contrast, LT through KCG film was relatively high, with ~60% transmission at 300 nm. In line with the latest result, Kassab et al. (2019) reported high light transmission in KCG‐based film. Moreover, they suggested that incorporating CNC (8 wt%) could reduce UV transmission to some extent (Kassab et al., 2019). Incorporating KCG into WPI led to an increase in the LT in the UV region (200 to 400 nm), which was below 20%. Furthermore, the LT of WC film in the visible region remarkably declined compared to WPI and KCG. To the best of our knowledge, consumers prefer packaging with high transparency so that food within a coating can be easily seen. In other words, LT between wavenumbers 380 and 700 should be over 85%. Thus, the control film can be considered a semi‐transparent film. However, different trends of LT were observed in films constituting RGA. Both RGA‐contained films declined in the UV region, with LT below 10%. The significant difference between control and RGA‐containing films was seen in the wavelength range between 400 and 600, covering the primary spectrum that eyes can detect, in a way that LT was 20%–40%, around 10%, and around 0% for control, 5% RGA, and 10% RGA films, respectively. In other words, the 10% RGA contained film was almost opaque to the naked eye. This could be ascribed to the absorption of UV–vis radiation by an aromatic ring in anthocyanins. The same phenomenon was confirmed by Kang et al. (2021), who reported that the LT was about 0 for alginate‐nanocrystalline cellulose containing red GSE. Like the RGA curve trend, the LT of RGA‐contained films continuously increased at wavelengths above 600 nm.

The results of the transparency test also confirmed the latest explanation (Table 4). More to the point, Tr600 for the control film (6.26) was significantly higher than those treatments containing 5% and 10% RGA, 3.99 and 1.83, respectively (*p* < .05). In agreement, Alizadeh‐Sani et al. (2021) observed that Tr600 of methylcellulose‐chitosan nanofiber significantly declined from 19.9 to 13.7 after the incorporation of barberry anthocyanin extract. In line with our results, other investigations proposed that as the concentration of anthocyanins increased, the films became less transparent (Pramitasari et al., 2022; Roy et al., 2021; Zhang et al., 2020). Therefore, it is necessary to incorporate sufficient anthocyanins in the films to ensure they possess desirable UV–vis light barrier properties and transparency.

**TABLE 4:** Antioxidant activity, antimicrobial properties, and transparency of WC and RGA‐contained films^†^.

Film	DPPH radical scavenging (%)	Inhibition zone (mm)			Tr_600_
		*Staphylococcus aureus*	*Escherichia coli*	*Candida albicans*	
WC	4.57 ± 0.86^c^	0.00	0.00	0.00	6.26 ± 0.07^c^
WCA5	68.77 ± 2.47^b^	3.50 ± 0.71^b^	0.00	0.00	3.99 ± 0.18^b^
WCA10	79.21 ± 3.02^a^	6.00 ± 1.41^a,A^	2.50 ± 0.71^B^	0.00	1.83 ± 0.18^a^

*Note*: Different letters in the same column indicate significant differences between treatments (*p* < .05).

Different letters in the same row (for the inhibition zone) indicate significant differences between treatments (*p* < .05).

Abbreviations: RGA, anthocyanins extracted from red grape pomace; WC, Whey protein isolate/κ‐carrageenan film; WCA10, WC film constituting 10 wt% RGA; WCA5, WC film constituting 5 wt% RGA.

^†^ All values are mean ± SD.

### 3.6 TGA

**FIGURE 3:**
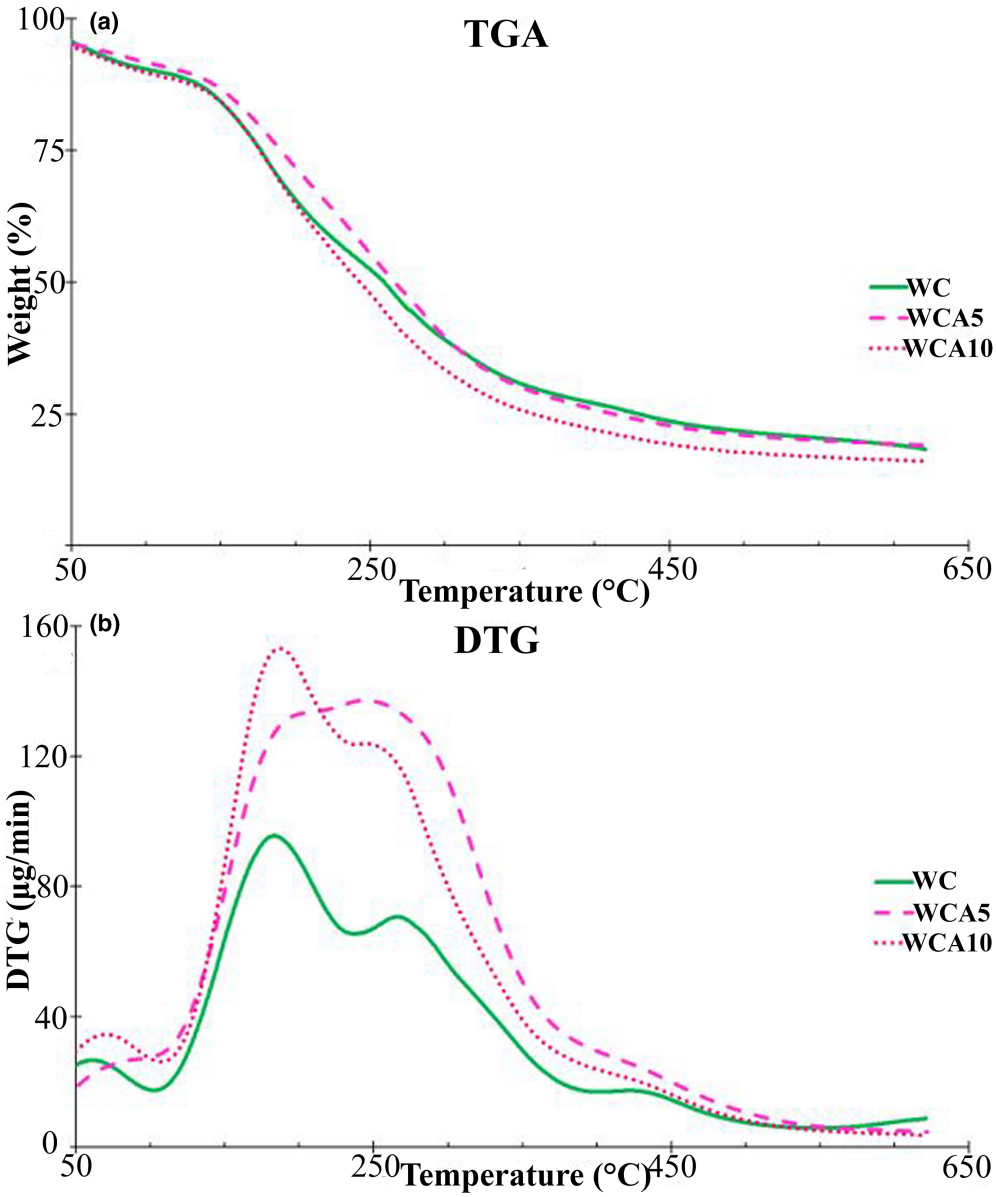
TGA (a) and DTG (b) thermographs of WC and RGA‐contained films. Abbreviations: RGA, anthocyanins extracted from red grape pomace; WC, Whey protein isolate (WPI)/κ‐carrageenan film (KCG); WCA5, WC film constituting 5 wt% RGA; WCA10, WC film constituting 10 wt% RGA.

Figure 3 demonstrates the TGA (a) and DTG (b) curves of WC, WCA5, and WCA10 films. The weight loss curve can be divided into three distinct regions, including Region I (50–110°C), region II (110–240°C), and region III (240–550°C), corresponding to the evaporation of free water molecules (~10%), degradation of low molecular weight compounds, RGA, and volatilization of bound water and glycerol (32%–40%), and degradation of the polymeric chain of KCG and WPI (34%–38%; Yekta et al., 2020). The 50% weight loss temperatures for WC, WCA5, and WCA10 films were 259.38°C, 266.28°C, and 243.04°C, respectively. Furthermore, the char at 550°C was 20.42%, 19.99%, and 16.85% for WC, WCA5, and WCA10 films, respectively. The latest findings suggested that the incorporation of 5% RGA might not significantly alter the heat‐resistant properties of the film. In line with this result, Wang et al. (2019) reported that adding red cabbage extract up to 15% did not significantly alter the thermal stability of *Artemisia sphaerocephala* Krasch gum/ carboxymethyl cellulose sodium film. However, an RGA dose at 10% could affect the thermal properties of the film, possibly due to interfering with the interactions between WPI and KCG in the film matrix. More to the point, the maximum weight loss temperature (T_M_) increased by incorporating 5 wt% RGA from 182.95 to 244.36. However, further increment in the concentration of RGA (WCA10 film) led to a decrease in the TM to 187.19. Likewise, some studies proposed the negative impact of anthocyanin incorporation on the heat stability of films (Prietto et al., 2017; Silva‐Pereira et al., 2015). To the best of our knowledge, anthocyanin loading content, polymer type, and the film‐forming solution's pH might affect anthocyanins' influence on the film's thermal properties (Freitas et al., 2020).

### 3.7 Antioxidant and antimicrobial properties

DPPH radical scavenging assessment determines films' antioxidant activity (AA; Table 4). Films' AA considerably increased from 4.57% to 68.77% and 79.21% by incorporating 5% and 10% RGA, respectively (*p* < .05). Native WC film presented a minor antioxidant activity, possibly due to some amino acids and peptides with antioxidant properties originating from WPI (Aluko, 2015). The latest observation also agrees with a previous study also showed that pristine WPI film possessed an antioxidant activity of 15.02% based on DPPH assay (Mohammadian, Moghaddam, Sharifan, et al., 2021). As the film in our study was a blend of two polymers (WPI and KCG), this could be the reason behind the decreased AA compared to native WPI film. It is widely believed that the extracts of red grape marc, either liquid or powder, can significantly increase the AA of films because of being a rich source of polyphenolic compounds, especially anthocyanins (Ciannamea et al., 2016; Dordevic et al., 2021). For instance, Dordevic et al. (2021) investigated the AA efficiency of chitosan films constituting red grape, blueberry, and parsley waste extracts. They demonstrated that RGA‐contained film had the highest AA, possibly due to higher anthocyanin content. In another study, the production of soy protein‐red grape extract films through the compression molding method led to higher AA than those prepared through casting (Ciannamea et al., 2016).

Food packaging films with antimicrobial properties are important to prevent pathogen growth and food spoilage, thus extending the shelf life of food products. The antimicrobial effect of films against some pathogenic microorganisms, including *S. aureus* (Gram +), *E. coli* (Gram–), and *C. albicans* (fungi), of food products was reported as inhibition diameters (Table 4). The control film (WC) did not show any antimicrobial properties. Our results suggest that the antibacterial properties of films significantly improved with a rise in the RGA concertation from 5 to 10 wt%. For instance, the inhibition zone of *S. aureus* significantly increased from 3.5 to 6.5 mm by raising the RGA dose from 5 to 10 wt%. Moreover, *S. aureus* was more susceptible than *E. coli* to RGA‐contained films. However, RGA was ineffective against *C. albicans*. In line with the latest results, several investigations revealed that anthocyanin‐rich films exhibit greater antimicrobial activity against Gram‐positive bacteria due to differences in cell wall structure, physiology, and metabolism compared to Gram‐negative bacteria (Wu et al., 2020; Zepon et al., 2019; Zhang et al., 2019). In contrast, a previous investigation suggested the higher antimicrobial activity of chitosan film containing the extract of red grape by‐product (RGE) against *E. coli* than *S. aureus* (Dordevic et al., 2021). This discrepancy could be due to the higher antibacterial activity of chitosan against gram‐negative bacteria (Chung et al., 2004; No, 2002). More to the point, the interaction between chitosan and RGE might boost its antibacterial activity against *E. coli*. Furthermore, the latest study reported that chitosan films containing the extracts of blueberry and parsley by‐products did not exhibit any antimicrobial activity against *E. coli, S. aureus*, and *C. albicans*. Overall, the antimicrobial activity of anthocyanin‐rich films depends on various factors, such as the origin and variety of the plant/fruit containing anthocyanin and the extraction and purification conditions (Katalinić et al., 2010; Lozano‐Navarro et al., 2017). These differences alter the composition and content of anthocyanins, which subsequently affect antimicrobial properties. Recent studies proposed the co‐incorporation of some antimicrobial substances, including essential oils and nanoparticles, and anthocyanins into films could considerably enhance the antimicrobial properties (Bakhshizadeh et al., 2023; Chen et al., 2020; Hao et al., 2023; Sun et al., 2020; Zhang, Cao, et al., 2023).

### 3.8 Microstructure

**FIGURE 4:**
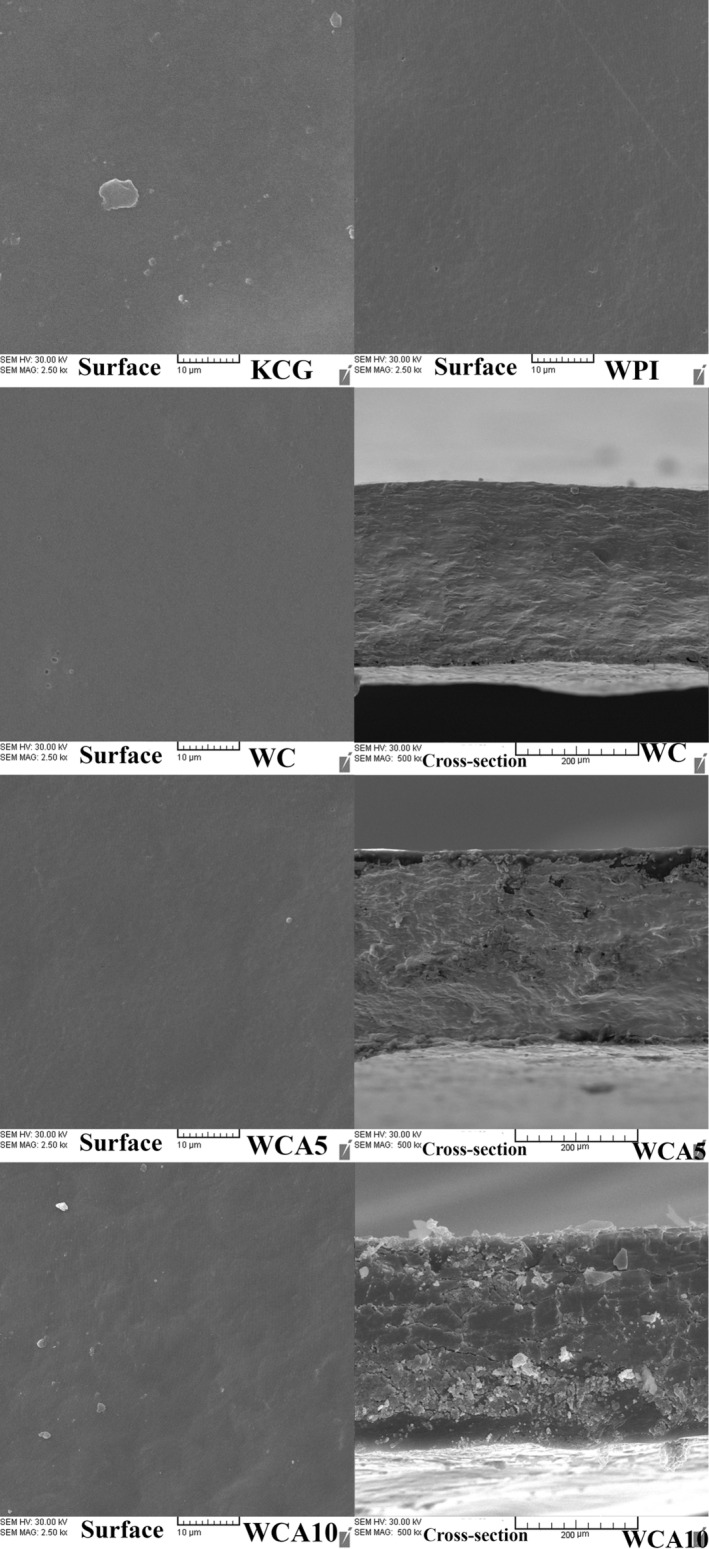
Surface (×2500 magnification) and cross‐section (×500 magnification) images of film treatments. Abbreviations: KCG, κ‐carrageenan; RGA, anthocyanins extracted from red grape pomace; WC, Whey protein isolate (WPI)/κ‐carrageenan film (KCG); WCA5, WC film constituting 5 wt% RGA; WCA10, WC film constituting 10 wt% RGA; WPI, whey protein isolate.

The arrangement of different film components and their impact on physical properties can be better understood through analysis of the films' microstructure. The surface image of all films presented a homogeneous structure without any substantial pores, cracks, or irregularities (Figure 4). However, by raising the RGA dose to 10 wt%, the surface of the latest treatment became slightly rough. The same observation was reported by (Kang et al., 2021) in gum arabic (GA)/ cellulose nanocrystals (CNC) film‐based film containing different fruit extracts. The cross‐section view of WC revealed that the interaction between WPI and KCG was compatible, leading to a smooth and compact microstructure. Similarly, a previous study reported that the film based on WPI and KCG at a 1:1 ratio showed a homogeneous and compact microstructure, whereas some micropores appeared at a 3:1 ratio (Sogut et al., 2022). By incorporating 5 wt% RGA, the cross‐sectional microstructure became slightly rougher, but no crack or pore was observed.

Further increment in RGA concentration to 10 wt% led to significant changes in microstructure, and some small cracks also appeared. The appearance of discontinuous regions with cracks, cavities, and aggregations distributed along the film matrix (cross‐section view of 10 wt% RGA treatment) of the film could be due to the entrapment of excess content of RGA particles in the continuous polymer network of film, which subsequently disrupted compact internal structure of the treatment (Liu et al., 2019). Furthermore, the impairment of barrier properties, that is, WVP and OTR, and mechanical properties of the film at 10 wt% RGA could be a result of reducing the film's homogeneity and compactness. In line with our results, Ma and Wang (2016), who incorporated GSE into tara gum/cellulose nanocrystal‐based film at 5, 10, and 15 wt%, detected increasing coarseness in the cross‐sectional image of 10 and 15 wt% films, which negatively affected the mechanical and O_2_ barrier properties of the films. Another study also observed the formation of some heterogeneous structures in cross‐section view after the incorporation of some fruit peel extract, including red pitaya, grape, and pomegranate, into GA/CNC film, which could be related to the aggregation of active ingredients of extracts in the film structure (Kang et al., 2021).

### 3.9 Moisture content and a_w_ of EFB

**FIGURE 5:**
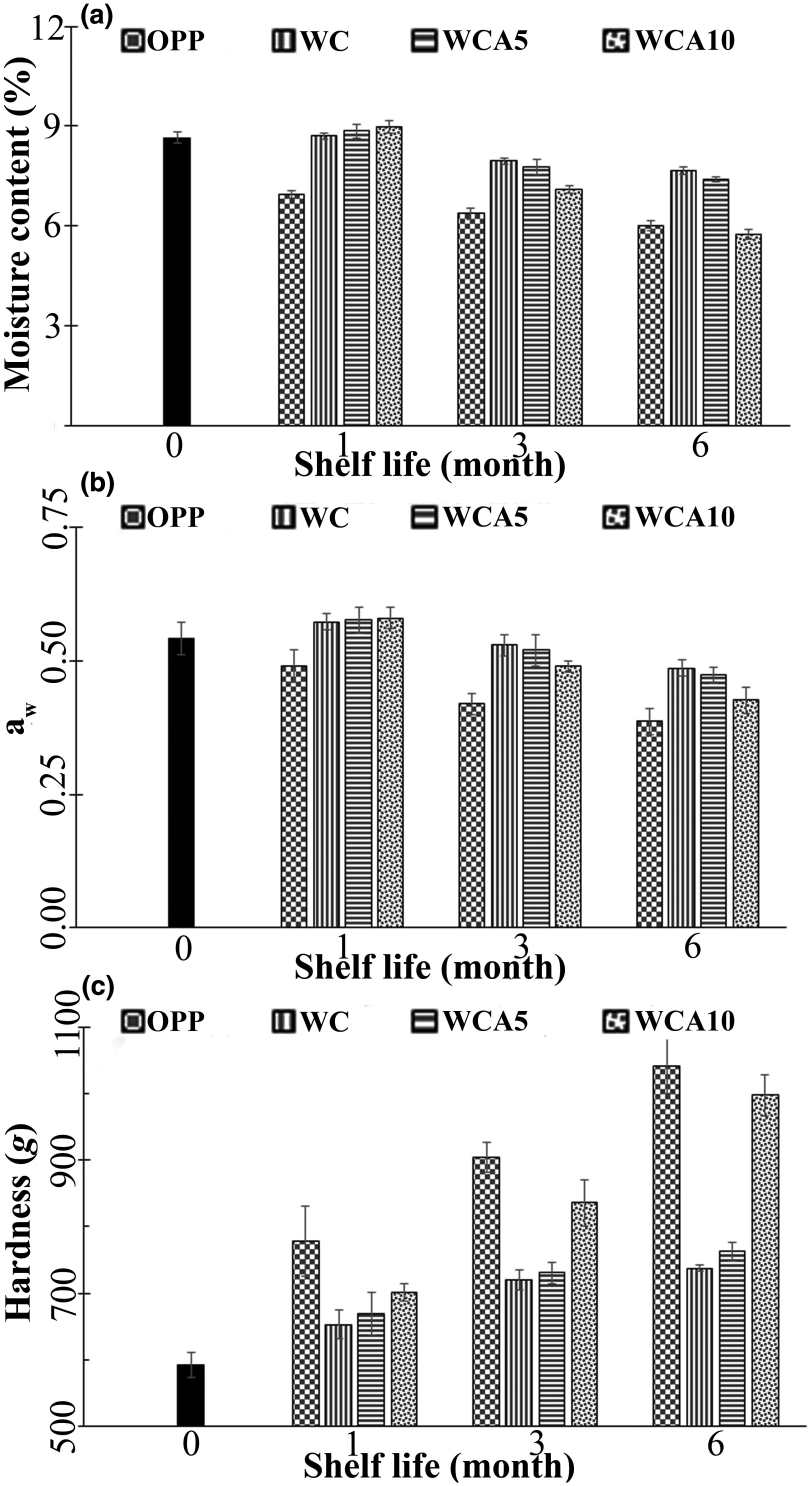
Moisture content (a), a_w_ (b), and hardness (c) of EFBs packaged by OPP, WC, WCA5, and WCA10 films during 6 months of storage at 38°C. Abbreviations: OPP, oriented polypropylene; WC, whey protein isolate/κ‐carrageenan film; WCA10, WC film constituting 10 wt% RGA; WCA5, WC film constituting 5 wt% RGA.

Figure 5 demonstrates moisture content (a) and a_w_ (b) of treatments coated with OPP, WC, WCA5, and WCA10 films during 6 months of storage at 38°C. MC after EFB production was 8.65%. Treatments showed varied trends during the shelf life period. In the first month, the MC of all treatments, except OPP, did not significantly change compared to day 0 (*p* > .05). Then, a reduction trend was observed for all treatments. At the end of the shelf life period, WC and WCA5 had the highest moisture contents, 7.68% and 7.41%, respectively. This indicated a moisture reduction of about 11%–14% compared to day 0. OPP and WCA10 showed higher moisture loss of approximately 30.59% and 33.63%, respectively. This implied that the WC and WCA5 films could effectively protect the EFB from moisture loss compared to the commercial OPP film. However, increasing the RGA dose to 10% impaired the film's barrier properties and efficiency in maintaining moisture content (Table 3). In general, the phenomenon known as moisture migration indicates the transfer of moisture from the interior areas of a product to its surface or crust, resulting in a reduction in overall moisture content and contributing to the acceleration of the staling process. In agreement, Hematian Sourki et al. (2021) stated that the cookies enriched with either lemon verbena powder or its essential oils demonstrated a considerable reduction in moisture content during storage. However, the powder increased moisture content due to constituting soluble fiber with a strong affinity toward water.

Water activity of EFB was 0.54 at day 0, and it did not significantly change for treatments coated by biodegradable polymers than OPP after the first month (*p* < .05). Then, from the third month until the end of storage, the a_w_ of all treatments significantly decreased (*p* < .05). The higher a_w_ reduction was for OPP (27.8%) and WCA10 (20.4%). On the other hand, WC and WCA10 treatments almost maintained their beginning a_w_ with a decrementation of only 9.3% and 13%, respectively (Figure 5b). It seemed that the film containing 10% RGA could not be an acceptable option for coating EFB to preserve moisture content. Generally, bakery products with water activity higher than 0.8 are microbiologically unstable (Suriya et al., 2017). On the contrary, the a_w_ of snack bars based on tapioca flour containing Brazilian nuts and fruits, including açaí and cupuassu, increased during the storage period, indicating that BOPP/metalized BOPP packaging did not provide an adequate barrier to prevent water vapor from permeating through it (Prazeres et al., 2020). Another study suggested that adding an edible layer between domains in a food system can minimize the changes in moisture and water activity of food ingredients (Suriya et al., 2017).

### 3.10 Hardness

The hardness of treatments coated with OPP, WC, WCA5, and WCA10 films is reported in Figure 5c. The hardness of EFB was 592.52 g immediately after cooking on day 0. Then, a gradual increase in hardness value was observed for all treatments. In the first month, the hardness of OPP, WC, WCA5, and WCA10 was promoted by 31.40%, 10.22%, 12.92%, and 18.31%, respectively. The latest trend continued to the end of the storage period, when the increment percentage reached 75.55%, 24.51%, 28.76%, and 68.37%, respectively. The hardness value indicates the product's freshness, which generally increases during the storage periods. To the best of our knowledge, the hardness of EFBs may be strongly influenced by several factors, including moisture content, baking time, and the content of specific components (sugar, shortening, and fibers; Mudgil et al., 2017). Therefore, the reduction of MC throughout the storage period might be the principal reason for the hardness increment. On the contrary, it was reported that the hardness of *Amorphophallus paeoniifolius* flour‐based cookies packaged by low‐density polyethylene (LDPE) gradually declined from 1.71 to 1.11 kg during a 21‐day storage period at 25°C. At the same time, the a_w_ of cookies increased, possibly due to moisture adsorption (Suriya et al., 2017).

### 3.11 Fatty acid rancidity

In order to precisely study the oxidation status of fatty acids during 6 months of storage at 38°C, peroxide value, p‐anisidine value, TOTOX value, and acid value were analyzed in the fat extracted from the EFB treatments in months 1, 3, and 6. Typically, PV measures the extent of oxidative breakdown in oils, fats, and foods containing fats. During the first month, a gradual increase in the PV of OPP and WC treatments was observed, whereas it did not significantly change in EFBs coated by antioxidant films (*p* > .05). In the third month, OPP and WC treatments reached the climax, representing PVs of 6.75 and 7.59 meq O/kg of fat, respectively. In comparison, it was revealed that the WCA5 and WCA10 films could effectively prevent fat from oxidation in a way that PV reached 2.93 and 3.37 meq O/kg of fat, respectively. In agreement with our results, it was observed that the PV increment rate decreased with increasing the incorporation percentage of clove powder from 0 to 2% in cookies stored at 28°C for 28 days (Aljobair, 2022).

The reduction trend of treatments coated by OPP and WC films could be ascribed to the degradation of hydroperoxides to secondary oxidation products, including aldehydes and ketones, which are more stable than hydroperoxides. It is widely believed that the analysis of just PV might not be an evident proof of the oxidative status of fats in food products. Therefore, the p‐AV was also measured to monitor the secondary oxidation products' production rate. The concomitant surveillance of both PV and p‐AV was recommended to better judge the fat oxidation status since they indicate the initial and final phases of oxidation of fatty acids. In the first month, the p‐AV of OPP and WC treatments slightly increased and remained under 3. On the other hand, WCA5 and WCA10 treatments showed no significant alteration in p‐AV compared to day 0. The highest increment in p‐AV occurred after 3 months of storage, and the value for OPP, WC, WCA5, and WCA10 reached a final point of 10.16, 11.82, 5.66, and 5.09, respectively. More to the point, the increment rate between months 1 and 3 for OPP, WC, WCA5, and WCA10 treatment was 33.87%, 37.18%, 24.68%, and 19.81% per month, respectively; in comparison, which was 40.73%, 43.49%, 24.77%, and 39.51% per month between months 3 and 6, respectively. The latest phenomenon could be attributed to increasing the decomposition of unstable hydroperoxides to relatively stable secondary oxidation products, especially during the third time interval (months 4–6). Similarly, a recent study investigated the effect of incorporating 4%–6% grape pomace powder into cookie formulation on the PV of the product during the first 2 months of the storage period (Theagarajan et al., 2019). They observed that 4% and 6% grape pomace could hamper the PV increment rate, and PV reached about 5.5 and 4.5 meq O/kg of fat, respectively, compared to the control treatment (~7 meq O/kg of fat) after 2 months of storage (Theagarajan et al., 2019). The latest observation implied that applying anthocyanin‐contained film on the product was more effective than directly incorporating the bioactive compounds into the product, possibly due to the thermal degradation of these components during cooking (Khanal et al., 2010). Similarly, Singh et al. (2020) reported that the level of secondary oxidation products and Free fatty acid content in the snack bars containing chia seed flour increased from 1.80 to 10.33 mg malonaldehyde/kg and 1.35% to 1.94% during a 28‐day shelf life period at 37°C. Moreover, the bars were more oxidatively stable in LDPE bags than cardboard boxes (Singh et al., 2020).

The TOTOX value demonstrates the level of carbonyl components produced in fats, along with other substances that result from the oxidation process that occurs during the shelf life of oils and fats. Therefore, it is a more comprehensive parameter than PV or p‐AV to estimate fatty acid oxidation during storage. The TOTOX curves of treatment confirmed the alteration trends in both PV and p‐AV curves of treatments. More to the point, the lowest TOTOX values were WCA5 and WCA10 treatments. OPP and WC treatments had the highest increment rates between months 1 and 3.

**FIGURE 6:**
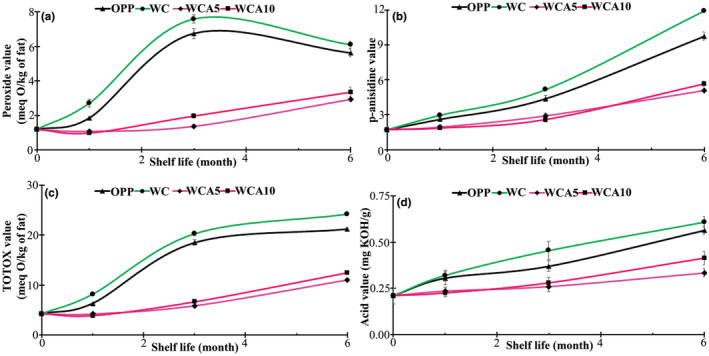
Peroxide value (a), p‐anisidine value (b), TOTOX (c), and acid value (d) of EFBs packaged by OPP, WC, WCA5, and WCA10 films during 6 months of storage at 38°C. Abbreviations: OPP, oriented polypropylene; WC, whey protein isolate/κ‐carrageenan film; WCA10, WC film constituting 10 wt% RGA; WCA5, WC film constituting 5 wt% RGA.

On the contrary, the highest TOTOX value increment rates for WCA5 and WCA10 treatments occurred from the third month to the end of the storage period. The acid value measures the amount of free fatty acid production due to the hydrolysis reaction of triglycerides. Throughout the storage period, Figure 6d revealed that acid value sharply increased in OPP and WC treatments with an accelerated speed in the first month of storage. Then, the rate of acid value increment gradually decreased until the end of storage. This could be due to reduced moisture content during storage, affecting the fat hydrolysis reaction. Generally, the higher the moisture content, the more the hydrolysis reaction rate accelerated. The analysis of curves related to WCA5 and WCA10 treatments revealed that both films could effectively hamper the hydrolysis reaction during the first month. Then, the acid value gradually increased until the end of the storage period, which might be aroused from the degradation of anthocyanins during the storage. Meethal et al. (2017) revealed that incorporating antioxidant jackfruit seed flour (JSF) into snack bars based on ragi flour (RF) led to declining free fatty acid (FFA) generation during 28‐day storage period at ambient temperature, especially in the formulations containing higher JSF:RF ratio. Moreover, the packaging type also influenced FFA content. For instance, those kept in metalized polyester film demonstrated a lower peroxidation rate than polypropylene‐coated snack bars, possibly due to higher oxygen permeation through polypropylene packaging film (Meethal et al., 2017).

Overall, the results of these analyses indicated that the fat oxidation stability was higher in EFBs coated by anthocyanin‐contained films than in OPP and WC films. The higher moisture content of WC‐coated EFB might be the reason for its poor oxidative stability compared to OPP treatment. The higher oxidative stability of films containing anthocyanin could be attributed to the potent antioxidant activity of anthocyanins (Hematian Sourki et al., 2021). A previous study also suggested the superior antioxidant activity of a solution containing 160 ppm of red grape marc extract (AA: 89.47%) than the same dose of BHT (AA: 87.14%; Negro et al., 2003). WCA5‐coated EFBs demonstrated slightly higher oxidative stability than WCA10‐coated ones, especially in the final month of the storage period, possibly due to the disruptive impact of the high dosage of RGA on some properties of WC film, especially its barrier properties. Furthermore, a more homogeneous film matrix might lead to the controlled release of bioactive components for longer (Falsafi et al., 2022). In agreement with the latest results, Hematian Sourki et al. (2021) revealed that the incorporation of lemon verbena essential oil into cookie formulation led to a considerable reduction in the increment rate of PV, p‐AV, TOTOX, and acid values during 6‐month storage, which was comparable to the treatment containing TBHQ as an antioxidant.

### 3.12 Sensory evaluation

**FIGURE 7:**
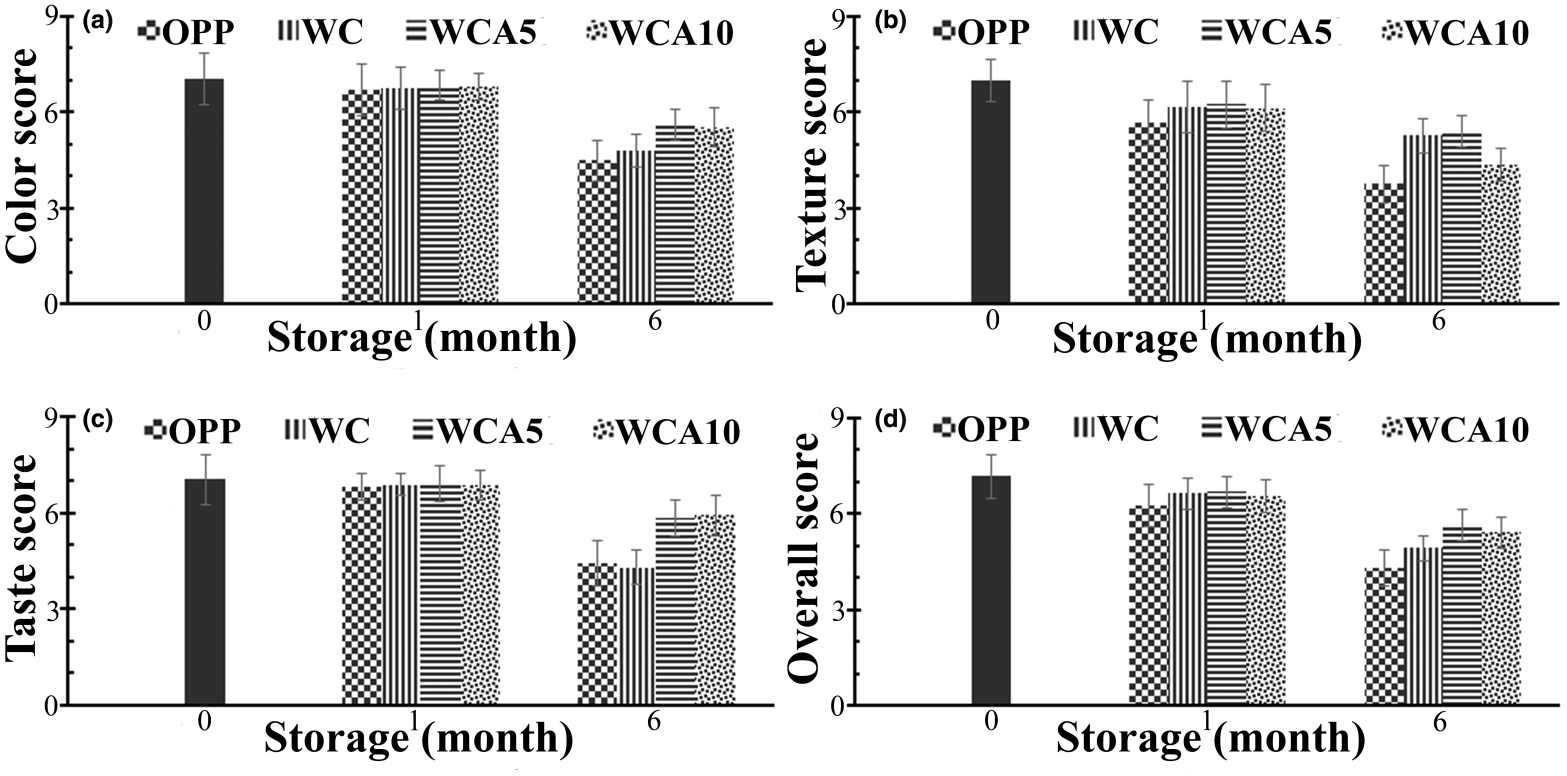
Color (a), Texture (b), Taste (c), and Overall acceptance (d) of EFBs packaged by OPP, WC, WCA5, and WCA10 films during 6 months of storage at 38°C. Abbreviations: OPP, oriented polypropylene; WC, whey protein isolate/κ‐carrageenan film; WCA5, WC film constituting 5 wt% RGA; WCA10, WC film constituting 10 wt% RGA.

Figure 7 illustrates the sensory evaluation results, including color, texture, taste, and overall acceptability, of EFB treatments during the storage period. Generally, the sensory parameters of all treatments significantly declined over the 6‐month shelf life period at 38°C (*p* < .05). Sensory results of color indicated that panelists scored 7.04 on day 0, and no significant alteration occurred in the color of the EFB treatments after the first month of storage compared to day 0 (*p* > .05). However, sensory panelists noticed differences in the color of EFBs coated by antioxidant film and those that did not. More to the point, they indicated that the latter looks pale and darker than those coated by the antioxidant film. Therefore, WCA5 and WCA10 treatments scored significantly higher than WC and OPP, especially at the end of 6 months of storage (*p* < .05). However, a significant reduction was observed between color scores of EFB treatments after the 6‐month storage compared to those of day 0 and the first month (*p* < .05). After 6 months of storage, the highest color score was related to the WCA5 treatment, obtaining 5.60. To the best of our knowledge, moisture content, temperature, and the permeability of packaging to O_2_ molecules are considered predominant factors impacting the color of bakery products during the shelf life period (Aljobair, 2022; Budžaki et al., 2014; Sharma, 2020; Suriya et al., 2017). Antioxidant films might have minimized color alteration by protecting the EFBs against oxidation reactions. It was observed that the direct addition of beetroot powder (BRP) to the snack bar formulation based on legumes and oil seeds led to turning its color to a slightly brown shade after a 90‐day storage period, possibly due to the deterioration of betacyanin in BRP. The effects of packaging type, including polyethylene, paper, and woven polypropylene bags, and storage temperature, −18, 25, 35, and 45°C, were investigated on the color of extrudates based on cereal and legume seeds during a shelf period of 90 days (Forsido et al., 2021). The best color liking was related to the treatment stored in polyethylene at −18°C. The color alterations in different treatments were claimed to be due to the enzymatic browning of polyphenols (Forsido et al., 2021; Hu et al., 2017).

The hardness score of EFB treatments insignificantly decreased after the first month of storage, except for OPP treatment, according to the opinions of sensory panels (*p* > .05). This was in line with the results of the hardness value. After 6 months of storage, the sensory panels concomitantly commented that the hardness of EFB treatments considerably increased, especially for OPP treatment. More to the point, the hardness score was 7.00 immediately after production, and it declined to 3.76 and 4.36 for OPP and WCA10 treatments at the end of the storage period, respectively. Other treatments gained significantly higher scores after 6 months (*p* < .05), which also correlated with the results of textural analysis (Figure 7c). The changes in the microstructure of the WCA10 film might lead to increasing WVP, which would consequently decrease the MC of EFB coated by WCA10 and improve the product hardness. In contrast, Rajagukguk et al. (2022) observed no significant reduction in the texture scores of granola bars based on chickpeas and green lentils sealed by LDPE during 2 months of storage at 20°C, and they reported that the aroma of the products was affected the most during the storage period. This discrepancy with the result of our study might be due to the differences in the product formulation, storage time, and temperature (Rajagukguk et al., 2022). Prazeres et al. (2020) developed snack bars based on tapioca flour containing Brazilian nuts and fruits. They reported that the texture scores of the invented bars significantly declined from 8 to <6. They reasoned that increasing the a_w_ of snack bars packaged in BOPP/metalized BOPP film was the leading cause of texture loss. Furthermore, they reported that the lowest likeness belonged to the texture scores compared to other sensory parameters, including appearance, color, and flavor (Prazeres et al., 2020). In fact, in the case of bakery products, it is essential to evaluate the texture, both instrumentally and sensorial, as it dramatically affects the overall quality of the product (Guiné, 2022).

The taste of EFB treatments slightly decreased after 1 month of storage. At this point, the highest score for taste belongs to WCA5 and WC10 treatments, obtaining 6.92 and 6.88, which was slightly lower than the scores on day 0 (7.28). It is noteworthy that sensory panels sensed fruity notes from EFBs coated by antioxidant films because of the grape taste of anthocyanin, and they liked it. After 6 months, the scores of EFB treatments considerably declined compared to day 0 and the first month. Furthermore, sensory panels gave lower scores to OPP (4.40) and WC (4.28) treatments than WCA5 (5.84) and WCA10 (5.92). This might be attributed to the production of off‐flavors as a consequence of the progressive oxidation of fats. More to the point, if the peroxide value of the fat contained within a given food product falls within the range of 10 to 20 meq O_2_ per kg, it is to be understood that the food has indeed undergone a state of rancidity. However, it is still deemed fit for consumption as its flavor and taste remain satisfactory. Conversely, should the value, as mentioned earlier, exceed 20 meq O_2_ per kg, the food product in question is no longer deemed acceptable for consumption by the consumer due to its strong off‐flavors. Since the fat content of the EFBs in our study was 14%, PV for the final products remained below 1.1 meq O_2_ per kg during storage. Another reason might be the oxidation of flavors of EFB during storage at 38°C. In other words, phenolic and aldehyde functional groups and aromatic nuclei are susceptible to oxygen (Weerawatanakorn et al., 2015). Similarly, another investigation reported that incorporating 16.5% BRP into snack bars based on legume and oil seeds led to significantly higher scores of color, appearance, texture, and taste compared to the treatment without BRP (Tangariya et al., 2022). They believed lipid peroxidation during 90 days of storage at either 5 or 15–35°C had been effectively hampered by BRP addition since the treatment without BRP was slightly rancid.

The overall acceptance of sensory panels gradually declined throughout the storage period. However, their scores demonstrated an insignificant decrementation in overall acceptance after the first month (*p* > .05). EFBs evaluated immediately after production scored 7.16, indicating the product was well‐liked. After 6 months, the overall acceptance declined to 4.32 and 4.92 for OPP and WC treatments, respectively. WCA5 and WCA10 treatments had significantly higher scores (*p* < .05) of 5.64 and 5.40, respectively. This suggests the antioxidant films can protect EFBs from undesirable sensorial changes during storage at 38°C.

## 4 CONCLUSIONS

Edible coatings and films are increasingly popular due to their affordability and reduced environmental impact. They are a sustainable technology that can be utilized to control gas exchange, moisture transfer, and oxidation in various products. Edible films and coatings can incorporate different active chemicals in food, enhancing safety, nutritional value, and sensory properties. In the bakery industry, edible films could create functional bakery products with added bioactive compounds, prebiotics, and probiotics. Food manufacturers have heightened efforts to extend shelf life and improve packaging technologies, protecting food from external variables and ensuring microbial safety. In the current study, the administration of antioxidant WC films containing RGA was found to be successful in decelerating fat oxidation reactions in EFBs during storage for 6 months at 38°C. The concentration of RGA predominantly impacted the properties of films. In other words, the incorporation of 10 wt% RGA led to impairing the mechanical, thermal, and barrier properties of films. In contrast, slight alterations occurred in the properties of WCA5 film in comparison with WC film. SEM images confirmed the tremendous structural changes in the cross‐section view of the WCA10 film. WCA5 and WCA10 films demonstrated high antioxidant activity and antibacterial properties against *S. aureus*, especially at 10 wt% RGA. Hydrogen bonding and electrostatic interactions might have been the main molecular forces maintaining RGA in the films' matrix. Throughout the storage at 38°C, EFBs coated by OPP, WC, WCA5, and WCA10 were tested for MC, a_w_, hardness, fat oxidation, and sensory evaluation on days 0 and months 1, 3, and 6. MC and a_w_ constantly declined during storage, with WC and WCA5 treatments more effectively maintaining the moisture within the packaging. Conversely, hardness gradually increased with the highest values recorded for OPP and WCA10 treatments. To monitor the oxidative rancidity of fats, PV, p‐AV, TOTOX, and acid values were measured, and the results suggested that OPP and WC treatments were more susceptible to oxidative reactions. Therefore, WCA5 and WCA10 treatments could effectively decline fat oxidation until the end of the shelf life period. Sensory assessment of EFB treatments demonstrated that the incorporation of RGA did not negatively affect the sensory properties of EFB treatments. Furthermore, sensory panels liked the fruity aroma sensed in the treatments coated by RGA‐contained films. WCA5 and WCA10 treatments gained the highest scores regarding overall acceptance. Consequently, WC films constituting 5% RGA can be a potential alternative for synthetic polymers to package foods containing high amounts of fats and susceptible to fat rancidity. The improved oxidation stability of EFBs indicated that the by‐products obtained from fruit and vegetable industries should be further explored for their promising applications in functional food development. Moreover, the co‐administration of the latest plant extract with other active ingredients such as metal–organic framework, nanomaterials, and essential oils may extend their application in different sectors of the food industry, for example, dairy and meat industries, to function as both active and intelligent packaging with improved functional properties.

## AUTHOR CONTRIBUTIONS

**Reza Yekta:** Conceptualization (equal); formal analysis (equal); investigation (lead); methodology (equal); software (equal); visualization (lead); writing – original draft (lead). **Arasb Dabbagh Moghaddam:** Conceptualization (lead); formal analysis (equal); project administration (lead); supervision (lead); writing – review and editing (lead). **Hedayat Hosseini:** Conceptualization (lead); data curation (lead); methodology (lead); supervision (equal); validation (lead); writing – review and editing (equal). **Anousheh Sharifan:** Formal analysis (equal); methodology (equal); software (equal). **Saeed Hadi:** Methodology (equal); resources (equal); software (equal). **Seyyed‐Javad Hosseini‐Shokouh:** Project administration (equal); resources (equal); supervision (equal).

## CONFLICT OF INTEREST STATEMENT

The authors hereby affirm that there are no conflicts of interest to declare.

## Data Availability

Data available on request from the authors.
